# Case Report: Two Case Reports of Pulmonary Hypertension after mRNA COVID-19 Vaccination

**DOI:** 10.3390/diseases11030114

**Published:** 2023-09-04

**Authors:** Robert D. Sullivan, Nataliia V. Shults, Yuichiro J. Suzuki

**Affiliations:** 1 U.S. Anesthesia Partners, Frederick, MD 21701, USA; 2 Department of Biology, Georgetown University, Washington, DC 20007, USA; ns1015@georgetown.edu; 3 Department of Pharmacology and Physiology, Georgetown University Medical Center, Washington, DC 20007, USA

**Keywords:** case report, COVID-19, mRNA, pulmonary hypertension, vaccine

## Abstract

Background: We herein report two cases of sudden onset symptomatic pulmonary hypertension after coronavirus disease 2019 (COVID-19) vaccination. Case Summary: Pulmonary hypertension in previously healthy adult males occurred within three weeks of receiving the second dose of the Pfizer (BNT162b2) mRNA COVID-19 vaccine from different lots. Both patients experienced a sudden onset of severe fatigue and dyspnea on exertion with negative severe acute respiratory syndrome coronavirus 2 (SARS-CoV-2) polymerase chain reaction (PCR) testing. The diagnosis was made by serial transthoracic echocardiography in the first case and by both transthoracic echocardiography and right heart catheterization in the second. Both cases resulted in functional limitations and likely permanent organ damage. No evidence of pulmonary emboli was detected in either case. Discussion: Pulmonary hypertension is a serious disease characterized by damage to lung vasculature and restricted blood flow through narrowed arteries from the right to left heart. The onset of symptoms is typically insidious, progressive and incurable, leading to right heart failure and premature death. The World Health Organization (WHO) classifies pulmonary hypertension into five categories and recently re-defined it as a resting mean pulmonary artery pressure greater than 20 mmHg. Sudden onset pulmonary hypertension would only be expected in the settings of surgical pneumonectomy or massive pulmonary emboli with compromise of at least 50% of the lung vasculature. We present here two novel cases of sudden onset pulmonary hypertension without evidence of pulmonary emboli, both of which occurred after receiving a COVID-19 mRNA vaccine.

## 1. Introduction

The pandemic of Coronavirus Disease 2019 (COVID-19) is caused by Severe Acute Respiratory Syndrome Coronavirus 2 (SARS-CoV-2). This virus enters human host cells through interactions between its viral membrane fusion protein, spike protein, and a host membrane protein, angiotensin-converting enzyme 2 (ACE2). In contrast to deaths from influenza, SARS-CoV-2 autopsy specimens demonstrated pulmonary artery (PA) wall thickening and vascular smooth muscle cell enlargement [1]. A meta-analysis showed that pulmonary hypertension is prevalent in COVID-19 patients [2]. Cases of pulmonary hypertension were found by echocardiography in 39% of COVID-19 intensive care unit (ICU) patients enrolled in April/May 2020 and were associated with mortality [3]. COVID-19 survivors experiencing fatigue and exercise limitations after hospital discharge were found to have pulmonary hypertension by right heart catheterization [4]. Recombinant SARS-CoV-2 spike protein S1 subunit alone is sufficient to elicit cell signaling in human lung vascular cells [1], raising the possibility that full-length spike protein from mRNA vaccines could also cause vascular remodeling and clinically significant pulmonary hypertension [5].

## 2. Case Description

We report here two cases of pulmonary hypertension after the second dose of the Pfizer mRNA COVID-19 vaccine (BNT162b2).

**Figure 1:**
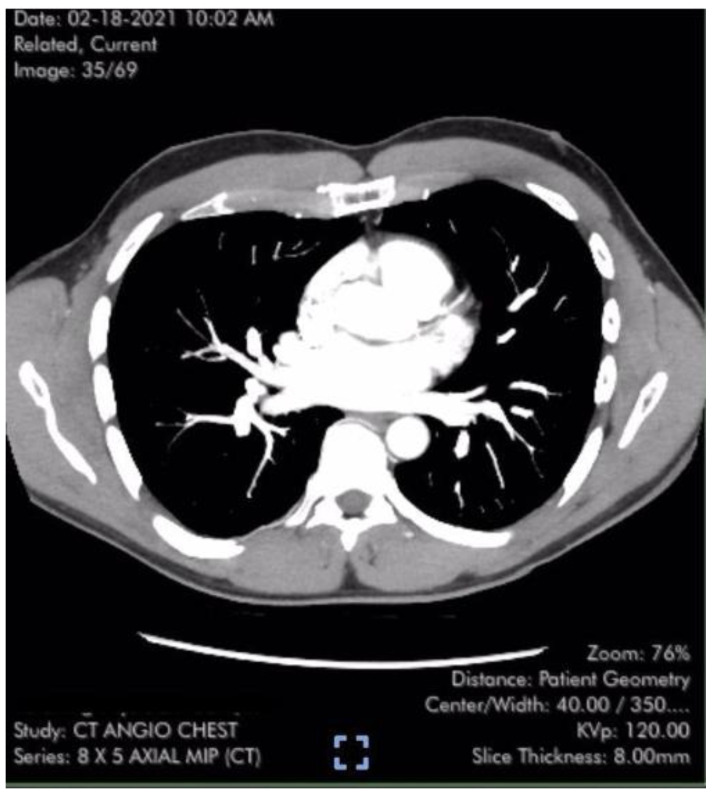
Axial CT image of the Case #1 patient from normal 3-dimensional pulmonary artery reconstruction without evidence of arterial clots or pulmonary emboli [6,7].

Case #1 is a previously healthy 49-year-old male physician athlete, body mass index (BMI) 23, non-smoker with a history of mild exercise-induced asthma treated with albuterol. The patient completed the primary series of Pfizer mRNA COVID-19 vaccine (BNT162b2), Dose 1 in December 2020 and Dose 2 in January 2021. Approximately three weeks after the second dose, the patient suddenly developed severe fatigue, flu-like symptoms, tachycardia, palpitations, orthostasis, right-sided chest pressure and dyspnea on exertion. SARS-CoV-2 polymerase chain reaction (PCR) testing was negative at the time of the onset of these symptoms. Transthoracic echocardiogram revealed normal left ventricular function with an ejection fraction (EF) of 65%, normal right ventricular size and function and a maximal tricuspid regurgitation velocity (TRV_max_) of 3.09 m/s. The estimated right ventricular systolic pressure (RVSP) of 42 mmHg was interpreted as mild/moderate pulmonary hypertension (Table 1). Laboratory studies including measurement of brain natriuretic peptide (BNP) (22 pg/mL; reference range < 900 pg/mL) were unremarkable except for elevated low-density lipoprotein (LDL) cholesterol and a hematocrit of 50%. Pulmonary computer tomography (CT) angiogram with 3D reconstruction of the PA tree was normal without evidence of pulmonary clots (Figure 1). The patient subsequently developed 15 lbs of fluid gain and generalized swelling, neck pressure, headaches and a feeling of “being hung upside down” consistent with jugular vein distention (JVD) and cerebral venous congestion. The resting oxygen saturation (SpO_2_) was 92% and there was new onset systolic and diastolic arterial hypertension. Symptoms and chest pressure occurred at rest and were exacerbated by exertion. Exercise and functional limitations were consistent with New York Heart Association (NYHA) Class 3–4. Serial echocardiograms showed no worsening of RVSP and continued normal RV function (Table 1). Symptoms and exercise tolerance improved to NYHA class 1–2 over one year. Fluid weight gain, swelling, tachycardia and arterial hypertension resolved and the resting SpO2 increased to 98–100%. Flu-like symptoms and fatigue diminished but did not disappear. RVSP remained elevated and essentially unchanged by follow up echocardiography (Table 1). This case was reported to the Vaccine Adverse Event Reporting System (VAERS ID 1039123).

Case #2 is a previously healthy and active 56-year-old male, BMI 25, non-smoker, with a history of spontaneous deep venous thrombosis (DVT) on two occasions which were resolved with courses of anticoagulation without symptoms of pulmonary emboli. Hematologic investigation identified no clotting abnormalities. In 2005, an incidental isolated left superior vena cava was suspected by an otherwise normal transthoracic echocardiogram and confirmed by cardiac magnetic resonance imaging (MRI). The patient completed the primary series of Pfizer mRNA COVID-19 vaccine (BNT162b2) Dose 1 and Dose 2 in April 2021. Twelve days after the second dose, the patient experienced sudden onset fatigue, flu-like symptoms and dyspnea on exertion. SARS-CoV-2 PCR testing was negative at the time of the onset of these symptoms. Exercise tolerance was consistent with NYHA Class 2. The patient sought medical attention two weeks later, but a stress echocardiogram and CT pulmonary angiography were not performed until almost 4 months after vaccination. The stress echocardiogram revealed normal left ventricular function, an ejection fraction (EF) of 60%, ventricular ectopy and mild right-sided chamber enlargement. No right-sided pressures were measured. A pulmonary CT angiogram with 3D reconstruction revealed mosaic attenuation of lung parenchyma with relative pruning of distal pulmonary vessels and mild enlargement of the PA without evidence of pulmonary emboli [8]. A Ventilation Perfusion (V/Q) scan was interpreted as near normal and a very low probability for pulmonary emboli. Despite the negative studies, anti-coagulation with rivaroxaban was started out of an abundance of caution due to patient’s distant history of DVT. A complete echocardiogram performed 5 months after vaccination measured a TRV_max_ of 2.82 m/s and calculated an RVSP of 40 mm Hg (Table 2), suggesting a diagnosis of pulmonary hypertension. Follow-up echocardiography three months later measured a TRV_max_ of 3.22 m/s and estimated an RVSP of 46 mmHg (Table 2). Subsequent right heart catheterization confirmed the diagnosis with directly measured systolic and diastolic PA pressures (PAP) of 44/18 mm Hg (mean 28 mm Hg) and elevated pulmonary vascular resistance (PVR) calculated at 3.6 Woods units. An endothelin receptor antagonist was prescribed and then a phosphodiesterase inhibitor. Fifteen months after vaccination, the patient’s exercise tolerance remained unchanged and consistent with NYHA Class 2 and the RVSP remained elevated at 49 mm Hg, estimated by a TRV_max_ of 3.22 m/s (Table 2). The patient’s course was complicated by transient episodes of new onset atrial fibrillation. BNP remained in normal range at 99 pg/mL. A subsequent Cardiac MRI revealed a mildly enlarged right ventricle with normal systolic function and normal main PA. A pulmonary hypertension screening panel by Invitae Genomics was negative for twelve genetic predisposition markers.

The clinical findings of the two cases are summarized in Table 3.

## 3. Discussion

We report here two cases of sudden onset pulmonary hypertension in the absence of pulmonary emboli and representing a COVID-19 mRNA vaccine (Pfizer BNT162b2) as a possible primary cause of pulmonary hypertension. The timing of 2–3 weeks after the second dose and flu-like symptoms suggests an immune-mediated mechanism.

While the definition of pulmonary hypertension is precise, the gravity of the diagnosis is difficult to convey. Even “mild” pulmonary hypertension is considered incurable. It is usually progressive and, for many, a terminal diagnosis. Pulmonary hypertension is best understood as a restriction in blood flow through the lung from the right ride of the heart to the left. The delivery of blood and oxygen to all organs is decreased, including the heart itself. This is especially so with exertion, as the right heart strains while the left ventricle remains underfilled and cannot increase cardiac output to meet metabolic demand. This is why pulmonary hypertension usually presents clinically and is experienced by the patient as shortness of breath with exertion and often right parasternal chest pressure. PAP is the product of cardiac output multiplied by PVR. With damage to and loss of functional pulmonary vasculature, PAP rises along with PVR for any given cardiac output. The right ventricle is very sensitive to the increasing PAP (afterload) with diminished stroke volume and cardiac output, increased energy consumption, and compromised blood flow to the right ventricle [9,10].

The time course of the onset of pulmonary hypertension is of critical importance. In almost all etiologies, the onset of pulmonary hypertension is gradual, with an insidious onset of fatigue and dyspnea on exertion as cardiac output falls. The right ventricle has time to hypertrophy and adapt, thus minimizing symptoms for the degree of pulmonary hypertension. As the symptoms are at first subtle, the diagnosis is often delayed. How much abrupt increase in PA pressure the right ventricle can tolerate is not precisely defined, though it may be inferred from two clinical scenarios in which this occurs: loss of pulmonary vasculature with surgical lung resection and occlusion of vasculature with pulmonary emboli. The pulmonary vasculature exists in such abundance that even removal of an entire lung with an immediate increase in PAP is highly survivable. This represents a loss of 45% of the vasculature for removal of the smaller left lung and 55% for the larger right. Peak systolic pulmonary pressure increased on average to 36 mmHg by echocardiography after a left pneumonectomy and 48 mmHg after a right. Thus, an entire lung can be removed and the diagnostic threshold for pulmonary hypertension may not be reached or minimally so [11]. It stands to reason that perhaps 50% or more of the pulmonary vasculature was acutely compromised in the cases we present. This approximates the diagnostic threshold for mild pulmonary hypertension by echocardiography and yet represents profound organ damage.

Pulmonary hypertension is not usually symptomatically mild when it occurs abruptly with massive pulmonary emboli. The right ventricle has no time to adapt. It poorly tolerates the increased afterload of a sudden increase in pulmonary pressure with precipitous drops in stroke volume and cardiac output [12]. Patients describe chest pressure and feelings of “impending doom” and frequently die. In the setting of acute pulmonary emboli, PA pressures only begin to rise when one-third of the pulmonary is vasculature is compromised. PA pressures (and mortality) increase with occlusion of up to about two-thirds of PA, as measured by angiography. A mean PAP of 40 mmHg and systolic of 60 mmHg is probably the very upper limit a previously normal right ventricle can pump, and only for a short time [13]. A compromise of more than two-thirds of the pulmonary vasculature is likely fatal.

Traditionally, direct measurement of PAP by right heart catheterization is required for formal diagnosis of pulmonary hypertension. The first patient did not confirm the diagnosis by right heart catheterization, but a false positive result is unlikely. Echocardiography provides an estimate of peak and mean PA pressures derived from the calculated RVSP with reasonable correlation to direct invasive measurement by right heart catheterization. While false positives and negatives are possible both with overestimation and underestimation of pressures, the positive predictive value for pulmonary hypertension with a TRV_max_ of >3.0 m/s is 90% [14,15,16].

The clinical improvement in the case without resolution of pulmonary hypertension likely represents right ventricular adaptation. The patient’s exercise capacity returned to above 10 METs in a 6 min walk test >500 m, a low BNP and counterintuitively, a high LDL cholesterol, which are predictors of increased survival with pulmonary hypertension [17,18,19,20].

Very recently, Nakagawa and co-workers [21] published a report of another case of a 63-year-old woman who suffered from acute pulmonary hypertension following the second dose of the Pfizer mRNA COVID-19 vaccine. The authors concluded that this case was due to microthrombus formation and the patient was successfully treated by anticoagulants. Thus, the mechanism of this case of acute pulmonary hypertension following COVID-19 vaccination seems different from the two cases presented here.

The mRNA COVID-19 vaccines produce the full-length spike protein [22]. Considering the earlier finding by Lei et al. [23] that the SARS-CoV-2 spike protein impairs endothelial cells of the pulmonary circulation, the pathogenic mechanisms of the cases presented here may involve the vascular effects of the spike protein. The spike protein has been proposed to contribute to the adverse effects of COVID-19 mRNA vaccines, resulting in various other pathological conditions [24,25].

## 4. Conclusions

In the present study, we report two cases of sudden onset pulmonary hypertension after receiving an mRNA COVID-19 vaccine (Pfizer BNT162b2) without evidence of pulmonary emboli. There may be many other cases of significant pulmonary vasculature damage after COVID-19 mRNA vaccination in which echocardiography still would be normal or sub-diagnostic and thus elude diagnosis. As BNT162b2 encodes for the SARS-CoV-2 spike protein, further investigations of the relationships between the spike protein and pulmonary hypertension are warranted [5,22].

## Figures and Tables

**Table 1:** Serial echocardiogram measurements of TRV_max_ and estimated RVSP of Case #1 patient.

Date	TRV_max_ (m/s)	RVSP (mmHg)
16 February 2021	3.09	42
24 February 2021	3.1	42
18 March 2021	3.0	39
21 April 2021	2.9	38
17 June 2021	3.1	42
15 February 2022	3.2	45

**Table 2:** Serial echocardiogram measurements of TRV_max_ and estimated RVSP of Case #2 patient.

Date	TRV_max_ (m/s)	RVSP (mmHg)
September 2021	2.82	40
December 2021	3.22	46
July 2022	3.22	49

**Table 3:** Summary of clinical findings of Cases #1 and #2.

	Case #1	Case #2
Gender	Male	Male
Age (yr)	49	56
BMI	23	25
Smoking	No	No
Vaccine	BNT162b2	BNT162b2
Vaccine Date (Dose 1)	December 2020	April 2021
Vaccine Date (Dose 2)	January 2021	April 2021
Onset of Symptoms	~21 days after Dose 2	12 days after Dose 2
SARS-CoV-2 PCR Test	Negative	Negative
TRV_max_ (m/s)	2.9–3.2	2.8–3.2
Estimated RVSP (mmHg)	38–45	40–49
BNP (pg/mL)	22	99
Evidence of Pulmonary Clots	None	None
NYHA Class	3–4	2

## Data Availability

Data are available upon request.
